# Evaluating the Chichewa version of the London Measure of Unplanned Pregnancy in Malawi: a validation update

**DOI:** 10.1186/s13104-021-05645-1

**Published:** 2021-06-10

**Authors:** Jennifer A. Hall, Judith Stephenson, Geraldine Barrett

**Affiliations:** Research Department of Reproductive Health, UCL Institute for Women’s Health, London, UK

**Keywords:** Pregnancy intention, Measurement, London Measure of Unplanned Pregnancy, Malawi, Psychometric, Validation, Chichewa

## Abstract

**Objective:**

To investigate the psychometric properties of the validated Chichewa version of the London Measure of Unplanned Pregnancy in a large representative community-based sample in Malawi, a low-income country. We collected data on pregnancy intention from a cohort of 4244 pregnant women in Malawi using the validated Chichewa version of the London Measure of Unplanned Pregnancy (LMUP). We evaluated the psychometric properties of the Chichewa LMUP using classical test theory and confirmatory factor analysis to re-assess the performance of items one and six, which had weaker performance in the original smaller, facility-based validation sample.

**Results:**

The Chichewa version of the LMUP met all pre-set criteria for validation. There are now nine validations of the LMUP in different low-and-middle-income countries, confirming the validity and applicability of the LMUP in these settings.

## Introduction

The London Measure of Unplanned Pregnancy (LMUP) is a psychometrically validated measure of pregnancy intention that was developed in the United Kingdom in the early 2000s [1, 2]. Using six questions, scored zero, one or two, it produces a score of zero-to-12, with higher scores indicating a more planned/intended pregnancy. Since its publication it has been translated and validated in diverse settings and populations around the world. As of May 2021 there are seventeen validated language versions across 14 countries, including nine low- and middle-income countries [2–15], with more in progress.

The original evaluation of the Chichewa LMUP in Malawi in 2013 found the measure to be acceptable to women and psychometrically valid [4]. The Cronbach’s α (a measure of internal consistency) was 0.78 (above the standard cut point of 0.7 [16]). Item-rest correlations (which should be > 0.2 [17]) were at least 0.7 for four of the six questions, was borderline for the preconception preparation question (0.16) and was low for the contraception question (0.05). Hypothesis testing confirmed construct validity and principal component analysis confirmed unidimensionality (the Eigenvalue of the first component was 3.1), albeit with a borderline second compenent (Eigenvalue 1.0) which mostly represented the contraception question (loading 0.99). A sensitivity analysis to assess the effect of the removal of the contraceptive item (item one) showed slightly improved performance of the measure but as the LMUP was not significantly adversely affected by its inclusion, and for the purposes of international comparability, tracking of trends, and future relevance, we recommended retaining it.

The LMUP includes two behavioural items: question one on contraception and question six on preconceptual preparations. There are lower levels of both contraceptive use and preconceptual preparations in Malawi, and also more generally in sub-Saharan African countries, compared with the United Kingdom and other developed countries. Despite this, the pattern of the relationship of the items of the LMUP has been remarkably stable across international contexts: items two-to-five most strongly correlated with the overall score; items one and six (the behaviour items) less strongly correlated; and all items positively correlated with each other. Given the importance of improving pregnancy intention measurement in low-and-middle-income countries, and in particular the call to provide evidence on the performance of the LMUP in such [18], we sought to re-examine the performance of the Chichewa LMUP using data from a large, representative community-based cohort study of pregnant women in Mchini District, Malawi [19]. We were particularly interested to review the performance of items one and six given their poorer performance in the original validation, which was conducted on a smaller, facility-based sample of 125 women.

## Main text

### Methodology

4244 pregnant women in Mchinji District, Malawi, completed the Chichewa LMUP between March 2013 and July 2014. Pregnant women were identified by a community-based surveillance system and were visited, consented and interviewed at home by one of 25 local data collectors. A full description of the recruitment and of the cohort has previously been published [19]. Mchinji District is a rural area, where most inhabitants are subsistence farmers with very low levels of education.

We evaluated the LMUP using Classical Test Theory, in keeping with its development [2] and the previous Chichewa validation [4]. Rates of missing data for each item were assessed, as high levels of missing data can indicate a problem with the understanding of acceptability of an item [20]. Item discrimination was assessed by examining the endorsement of item response options. Internal consistency was assessed using the Cronbach’s α with a cut-point of 0.7 [16] and examination of each question’s item-rest correlation, accepting a minimum correlation of 0.20 [17]. Inter-item correlations were assessed to check they were all positive. To assess structural (construct) validity, we conducted a principal component analysis (PCA) looking for one component with an Eigenvalue larger than one to demonstrate that all items are measuring the same construct [21]. In keeping with recent recommended standards of assessment [22], confirmatory factor analysis (CFA) was carried out to assess model fit (in this case the six items to a unidimensional model). Model fit was assessed by the comparative fit index (CFI), with > 0.95 indicating acceptable model fit, and standardised root mean squared residual (SRMR), with < 0.08 indicating acceptable model fit. We also conducted a Mokken analysis, as other validations of the LMUP have, to confirm that the items vary in ‘difficulty’ and that women answered the LMUP questions in keeping with how planned their pregnancy was. A Loevinger H coefficient of > 0.5 indicates a ‘strong’ scale [23].

All analyses were conducted in STATA version 15 (StataCorp. 2017. Stata Statistical Software: Release 15. College Station, TX: StataCorp LLC).

### Results

Missing data were very rare, with only 19 missing answers out of 25 464 questions asked (the six LMUP questions asked of 4244 women), as shown in Table 1, suggesting extremely good acceptability. No question response had more than 80% endorsement. For questions one to five the more planned option was the most frequently endorsed, whereas for question six, preconception preparations, a lack of preparation was most common.

**Table 1:** Participant’s responses to each of the six London Measure of Unplanned Pregnancy questions

Response to LMUP questions	Freq	Percent
Q1—Contraception		
Always used contraception	230	5.42
Sometimes used contraception/knew the method failed	1187	28.0
Not using contraception	2823	66.5
Missing	4	0.09
Total	4244	100
Q2—Timing		
Wrong time	1460	34.6
Ok, but not quite right time	431	10.2
Right time	2344	55.2
Missing	1	0.02
Total	4244	100
Q3—Intention		
Did not intend to get pregnant	1628	38.4
Intentions kept changing	310	7.30
Intended to get pregnant	2304	54.2
Missing	2	0.05
Total	4243	100
Q4—Desire		
Did not want to have a baby	1438	33.9
Mixed feelings about having a baby	462	10.9
Wanted to have a baby	2344	55.2
Total	4244	100
Q5—Partner discussions		
Never discussed having children together	970	22.9
Discussed having children, but had not agreed to get pregnant	1011	23.8
Agreed we would like me to be pregnant	2261	53.3
Missing	2	0.05
Total	4244	100
Q6—Preconception preparation		
No action to prepare for pregnancy	2710	63.9
1 action to prepare for pregnancy	1055	24.9
≥ 2 actions to prepare for pregnancy	469	11.1
Missing	10	0.24
Total	4244	100

The full range of LMUP scores from zero to twelve was captured. The median score was nine, inter-quartile range three-to-11. The Chronbach’s alpha was 0.89 and, as shown in Table 2, all item-rest correlations were above 0.2. Principal components analysis confirmed that all items loaded on to one component with an Eigenvalue of 3.95. The Mokken analysis showed the LMUP was a ‘strong’ scale, with an overall Loevinger H coefficient of 0.733. The CFA confirmed a single factor model (CFI 0.997, SRMR 0.015).

**Table 2:** Results of classical test theory, principal component, Mokken and confirmatory factor analyses

	N	Item-rest correlation coefficient	PCA component 1 loading	Loevinger H coefficient	CFA factor loadings
Q1—Contraception	4240	0.283	0.185	0.320	0.285
Q2—Timing	4243	0.878	0.469	0.791	0.930
Q3—Intention	4242	0.899	0.478	0.816	0.959
Q4—Desire	4244	0.891	0.473	0.799	0.942
Q5—Partner discussions	4242	0.809	0.446	0.770	0.846
Q6—Preconception preparation	4234	0.504	0.310	0.755	0.507

### Discussion

In this large representative community dataset, from a rural area in Malawi, a low-income country, the psychometric properties of the Chichewa LMUP met all prespecified criteria and international standards. In particular, the item-rest correlations for questions one and six, i.e. the behavioural components of contraceptive use and preconception preparations, were higher than in the initial validation and were both above the cut-point of 0.2 [4]. The pre-to-post birth stability of the Chichewa LMUP has previously been investigated in this sample [24]. This showed that the LMUP has moderate to substantial stability between pregnancy and up to 12 months postpartum (AC 0.54–0.64).

Since these data were collected there has been one other assessment of the psychometric properties of the Chichewa LMUP in Malawi by Yeatman and Greenaway [25]. In their study of 645 women, the Chichewa LMUP also demonstrated excellent psychometric properties: Chronbach’s alpha = 0.86; all item-rest correlations > 0.2, including the contraception and preconception questions; principal components analysis demonstrating one component/unidimensional measurement; and a full range of scores with a bimodal distribution.

Hence, there have now been three analyses of the Chichewa LMUP in independent samples in Malawi, with the two more recent and larger studies demonstrating good psychometric properties. In addition to this, there are now 12 validationated language versions in a further eight low-or-middle-income countries: Uganda [14]; Sierra Leone [13]; Sri Lanka [12]; Pakistan [9]; India [6]; Iran [7]; Turkey [15] and Brazil [3]. The authors are aware of ongoing evaluations in Nepal, Botswana, Mexico, Tanzania, Mozambique, South Africa, India and elsewhere. In 2015, the Population Council’s Expert meeting on ‘Conceptualizing and Measuring Unintended Pregnancy and Birth’ recognised that the LMUP overcame many of the limitations of previous assessments of unplanned pregnancy but were concerned that, at that time, there was limited evidence of its validity outside high-income settings [18]. This concern is no longer founded. There is also growing evidence of the limitations of other methods, such as the Demographic and Health Survey questions which have recall bias and are affected by maternal characteristics and pregnancy outcomes, which the LMUP overcomes [24, 26–30].

## Conclusions

This large community-based dataset confirms the validity of the LMUP in a rural, low-income country setting. Furthermore, the item-rest correlations, coefficient and factor loadings for the contraception and preconception preparation questions demonstrate that these items are relevant in this context in all the analyses conducted. Given this, and the accumulating validations of the LMUP in other low- and middle-income countries, there is now convincing evidence of the validity of the LMUP, and the relevance of questions on contraception and preconception preparation, in diverse settings around the world.

## Limitations

The main limitation of this study is that, as women were recruited on average in the fifth month of pregnancy, we missed abortions and early miscarriages. Despite this, we captured the full range of pregnancies intentions (scores from zero to 12).

## Data Availability

The dataset supporting the conclusions of this article is available from the UCL Discovery database linked to the publication record in the UCL Research Publication Service. The dataset can be accessed here: http://www.homepages.ucl.ac.uk/~uccaags/00/6.html (https://doi.org/10.5522/00/6). Data from the study are also available from the corresponding author who may be contacted at jennifer.hall@ucl.ac.uk. The Chichewa LMUP is available at www.lmup.org.uk/docs/Chichewa_LMUP.docx

## Abbreviations

CFA: Confirmatory factor analysis
CFI: Comparative fit index
LMUP: London Measure of Unplanned Pregnancy
PCA: Principal components analysis
SRMR: Standardised root mean squared residual
